# Risk Factors for Empiric Treatment Failure in US Female Outpatients with Uncomplicated Urinary Tract Infection: an Observational Study

**DOI:** 10.1007/s11606-024-09029-6

**Published:** 2024-10-02

**Authors:** Debra L. Fromer, Meghan E. Luck, Wendy Y. Cheng, Malena Mahendran, Wilson L. da Costa, Megan Pinaire, Mei Sheng Duh, Madison T. Preib, Jeffrey J. Ellis

**Affiliations:** 1 Urology, Hackensack University Medical Center, Hackensack, NJ USA; 2 US Medical Affairs, GSK, Collegeville, PA USA; 3 Health Economics and Outcomes Research, Analysis Group, Inc., Boston, MA USA; 4 Global VEO, GSK, Collegeville, PA USA; 5 US VEO, GSK, Collegeville, PA USA

**Keywords:** Uncomplicated urinary tract infection, Treatment failure, Empiric prescription, Oral antibiotics, Risk factors

## Abstract

**Background:**

Treatment failure (TF) in uncomplicated urinary tract infection (uUTI) increases disease burden and risk of antimicrobial resistance. Identification of risk factors for TF could inform empiric treatment decisions and reduce suboptimal outcomes.

**Objective:**

To evaluate the incidence of TF to empirically prescribed oral antibiotics and identify risk factors for TF in females with uUTI in the United States (US).

**Design:**

This retrospective cohort study used Optum’s de-identified Electronic Health Record dataset (January 2017–September 2022).

**Patients:**

Eligible female patients aged ≥ 12 years had ≥ 1 diagnosis of urinary tract infection (UTI) in an outpatient ambulatory/emergency department (ED) setting, ≥ 1 empiric oral antibiotic prescription, and no evidence of complicated UTI (cUTI).

**Main Measures:**

TF was defined as having a new/repeat oral antibiotic prescription, IV antibiotic administration or acute UTI diagnosis ≤ 28 days following initial empiric oral antibiotic prescription​. Risk factors of TF were selected using LASSO and reported using adjusted risk ratios (aRR) and 95% CIs.

**Key Results:**

Of 376,004 patients with uUTI, 62,873 (16.7%) experienced TF. Incidence of TF was highest in patients with history of antibiotic TF (33.9%) or fosfomycin prescription (30.1%). Significant risk factors of TF included ≥ 3 prior antibiotic prescriptions (aRR [95% CI]: 1.60 [1.56–1.64]); fosfomycin prescription (1.60 [1.38–1.86]); uUTI diagnosis in ED (1.49 [1.46–1.52]), Southern US residence (1.37 [1.35–1.40]), age ≥ 75 years (1.35 [1.29–1.41]), recurrent UTI (1.12 [1.10–1.14]) and obesity (1.06 [1.04–1.08]).

**Conclusions:**

Incidence of TF to empirically prescribed oral antibiotics for uUTI is considerable. Prior infections requiring antibiotic prescription and location of care are key risk factors for TF in female outpatients with uUTI. Knowledge of these TF risk factors can inform shared-decision making and supplement existing guidance on uUTI treatment.

**Supplementary Information:**

The online version contains supplementary material available at 10.1007/s11606-024-09029-6.

## INTRODUCTION

More than half of females experience ≥ 1 urinary tract infection (UTI) in their lifetime^1,2^ and uncomplicated UTIs (uUTI) are among the most frequently occurring infections in the United States (US).^3^ The annual incidence of uUTIs is upwards of 1,500 cases per 100,000 females aged ≥ 15 years.^4^ UTIs are often treated empirically, which may lead to suboptimal outcomes, including increased risk of antimicrobial resistance (AMR) and treatment failure (TF), when inappropriate antibiotics are prescribed.^5–8^ In the US, 0.3–17% of patients with uUTIs experience TF,^5,9–12^ with varying incidence largely driven by TF definition heterogeneity. Incidence rates approaching 20% are notable as TF is associated with increased levels of UTI-related burden, including greater activity impairment and higher direct and indirect healthcare resource use.^10–13^ Patients with uUTI who experience antibiotic TF cost $570 more on average in outpatient visit expenditures compared to those without TF.^10,11^

To date, there is a dearth of research assessing risk factors for antibiotic TF in uUTI. In one randomized clinical trial (RCT), treatment with fosfomycin and age ≥ 52 years were associated with clinical failure, with the latter characteristic also associated with microbiological failure.^14^ An enhanced understanding of risk factors for TF could help optimize empiric antibiotic prescribing in uUTI and reduce the burden associated with TF. The Infectious Diseases Society of America (IDSA) guidelines for empiric treatment in uUTI recommend considering community-level thresholds of AMR when antibiogram data are available.^8,15^ However, these data are often unavailable as urine culture collection is not standardized across clinics or recommended by guidelines for most cases of uUTI.^15^ Therefore, identification of risk factors of TF that can be readily assessed in clinical practice is imperative to help inform empiric antibiotic prescribing.

To advance the understanding of TF in uUTI, this study assessed the incidence of TF to empirically prescribed oral antibiotics and identified risk factors for TF in female outpatients with uUTI in the US.

## METHODS

### Data Source and Study Design

This retrospective observational cohort study used Optum’s de-identified Electronic Health Record dataset from January 1, 2017, to September 30, 2022. As data were de-identified and compliant with the Health Insurance Portability and Accountability Act, no institutional review board approvals were required.

The study design is presented in Figure S1. The index date was defined as the date of the first empirically prescribed oral antibiotic ± 5 days of a randomly selected diagnosis of UTI (index uUTI diagnosis). The 5-day window was used in accordance with prior studies^10,11,16,17^ and to allow for the typical maximum length of an antibiotic regimen,^8^ a confirmatory diagnostic visit after antibiotic initiation, and any delay in medical record documentation. The baseline period was defined as the 12-month period before the index date, during which patient demographics, clinical characteristics and microbiology-related characteristics were described. The observation period was defined as the 28-day period after the index date,^10,11,16,18,19^ during which the incidence of oral antibiotic TF was assessed.

### Study Population

Eligible female patients were aged ≥ 12 years with ≥ 1 index uUTI diagnosis in an outpatient ambulatory/emergency department (ED) setting, ≥ 1 prescription for an oral antibiotic ± 5 days of the index uUTI diagnosis, and ≥ 12 months of Optum EHR activity pre- and post-index date. See the supplementary materials for complete information on the eligibility criteria.

### Study Outcome: TF

TF was defined as having ≥ 1 of the following outcomes ≤ 28 days after the index date:​ new/repeat oral antibiotic prescription; administration of an intravenous (IV) antibiotic; or acute UTI diagnosis, defined as a primary diagnosis of uUTI/cUTI in an ED/inpatient setting (excluding the index uUTI diagnosis).

Patients with or without TF were identified based on the observation or absence of TF ≤ 28 days post-index date, respectively.

### Baseline Characteristics

A full description of the baseline characteristics evaluated in this study is provided in the supplementary materials.

### Statistical Analysis

Baseline characteristics were compared between patients with/without TF using standardized differences (std diff), with differences > 10% indicating a meaningful imbalance.^20,21^

The incidence of TF was reported as a number and proportion of the total study population and by select baseline characteristics. A sensitivity analysis was conducted to assess the robustness of the definition of TF by evaluating its incidence using a more conservative definition (see supplementary materials).

Risk factors for TF were selected from a candidate list using least absolute shrinkage and selection operator (LASSO)-penalized regression and the magnitude of effect was reported using adjusted risk ratios (aRRs), 95% CIs, and *p*-values estimated from Poisson regression models with robust standard errors.^22^ Multicollinearity was assessed as part of the regression model building. The LASSO-penalized regression was conducted using R Studio, Version2022.02.3 (Posit Software, PBC, Boston, MA) and all other analyses were conducted using SAS Enterprise Guide, Version 7.15 (SAS Institute, Cary, NC).

## RESULTS

### Characteristics of Patients with/without TF

**Figure 1:**
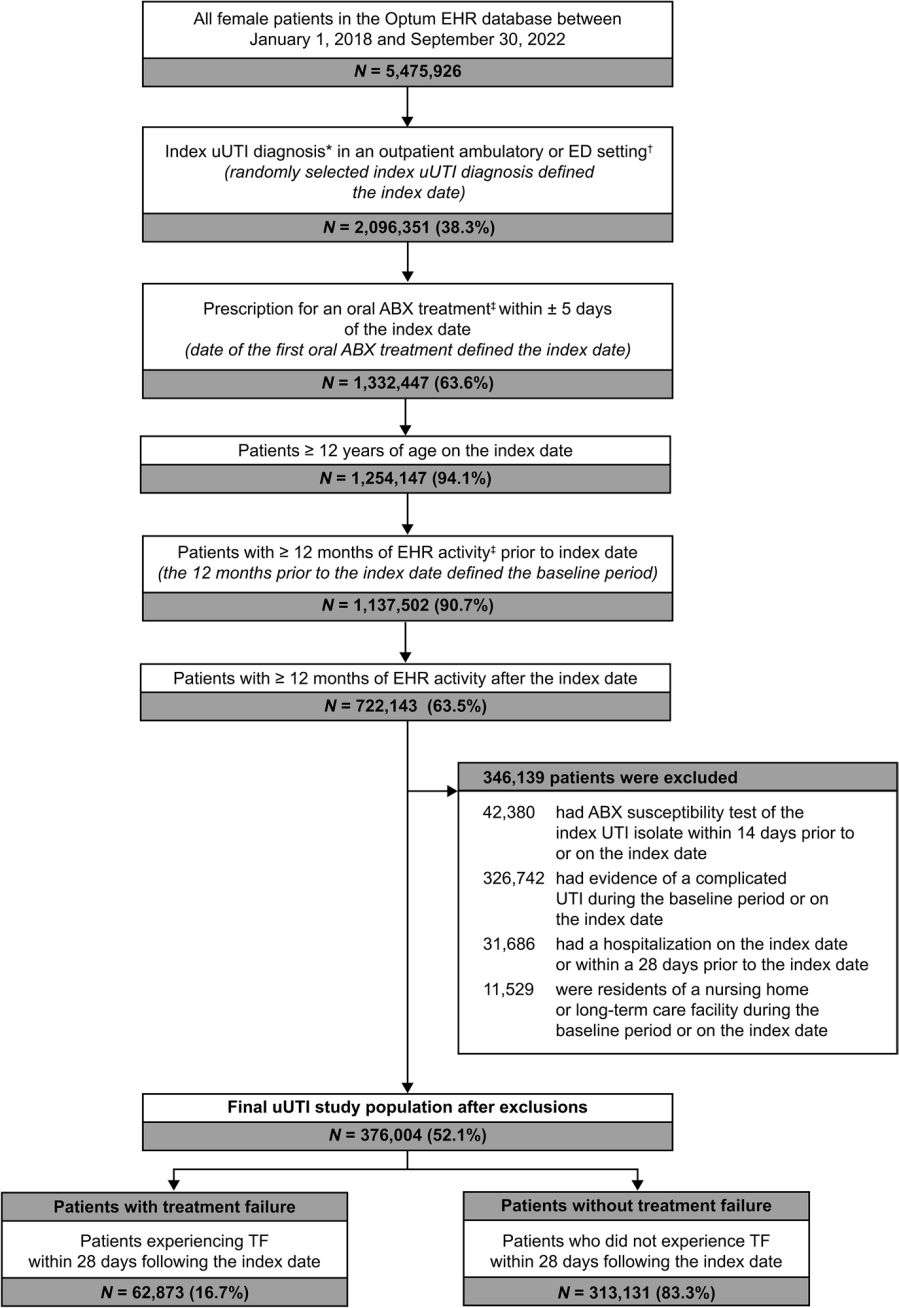
Patient Selection Flowchart. ABX, antibiotic; ED, emergency department; EHR, electronic health record; ICD-10-CM, international classification of diseases, tenth edition, clinical modification; NTF, nitrofurantoin; SXT, trimethoprim-sulfamethoxazole; TF, treatment failure; UTI, urinary tract infection; uUTI, uncomplicated urinary tract infection.

Overall, 376,004 female outpatients with uUTI were included in the study sample, of whom 62,873 (16.7%) experienced TF (Fig. 1). Patients with and without TF had a mean (SD) age of 48.9 (19.6) years and 46.5 (19.1) years (std diff 12.3%), respectively; were predominantly White and from the Midwest (Table 1).

**Table 1:** Incidence of TF, Stratified by Demographics of Female Outpatients with uUTI Failing or not Failing Empirically Prescribed Oral Antibiotics

Characteristics	Patients with TF (*n* = 62,873 cases)	Patients without TF (*n* = 313,131 cases)	Std diff (%)*	Incidence of TF (%)
Age at index, years, mean ± SD [median]	48.9 ± 19.6 [50]	46.5 ± 19.1 [47]	12.3*	N/A
Age categories, *n* (%)				
12–17	2163 (3.4)	12,544 (4.0)	3.0	14.7
18–34	15,382 (24.5)	86,585 (27.7)	7.3	15.1
35–49	13,531 (21.5)	71,691 (22.9)	3.3	15.9
50–64	17,066 (27.1)	82,386 (26.3)	1.9	17.2
65–74	7816 (12.4)	33,776 (10.8)	5.1	18.8
≥ 75	6915 (11.0)	26,149 (8.4)	9.0	20.9
US region, *n* (%)				
Midwest	32,891 (52.3)	170,983 (54.6)	4.6	16.1
South	14,437 (23.0)	49,976 (16.0)	17.8*	22.4
Northeast	6338 (10.1)	39,402 (12.6)	7.9	13.9
West	6216 (9.9)	37,231 (11.9)	6.4	14.3
Other/unknown^‡^	2991 (4.8)	15,539 (5.0)	1.0	16.1
Race, *n* (%)				
White	51,563 (82.0)	252,935 (80.8)	3.2	16.9
Black	6671 (10.6)	31,228 (10.0)	2.1	17.6
Asian	1099 (1.7)	6379 (2.0)	2.1	14.7
Unknown	3540 (5.6)	22,589 (7.2)	6.5	13.5
Ethnicity, *n* (%)				
Not Hispanic	55,103 (87.6)	270,563 (86.4)	3.7	16.9
Hispanic	3863 (6.1)	19,144 (6.1)	0.1	16.8
Unknown	3907 (6.2)	23,424 (7.5)	5.0	14.3
Insurance plan type, *n* (%)				
Commercial	34,023 (54.1)	183,058 (58.5)	8.8	15.7
Dual enrollment^§^	12,927 (20.6)	52,843 (16.9)	9.5	19.7
Medicare	6738 (10.7)	29,385 (9.4)	4.4	18.7
Medicaid	4723 (7.5)	27,064 (8.6)	4.2	14.9
Uninsured	2243 (3.6)	9929 (3.2)	2.2	18.4
Other	852 (1.4)	4035 (1.3)	0.6	17.4
Unknown	1367 (2.2)	6817 (2.2)	0.0	16.7

^*^A std diff > 10% was considered a clinically meaningful imbalance between patients with TF than those without TF

^‡^Any region that did not fall under one of the four US census bureau regions in the Optum EHR dataset

^§^Enrollment in ≥ 2 types of insurance plans on the index date

EHR, electronic heath record; N/A, not applicable; SD, standard deviation; Std diff, standardized difference; TF, treatment failure; US, United States; uUTI, uncomplicated urinary tract infection

Among patients with/without TF, nitrofurantoin (NTF) was the most frequently prescribed antibiotic on the index date (34.2% vs. 38.0%; std diff 8.0%). During the baseline period, more patients with TF had ≥ 3 prescriptions for an oral antibiotic (18.5% vs. 11.3%; std diff 20.4%), recurrent UTI (22.6% vs. 17.0%; std diff 14.1%), and history of antibiotic TF (12.1% vs. < 5%; std diff 26.8%; Table 2) than patients without TF.

**Table 2:** Incidence of TF, Stratified by Clinical and Microbiology-related Characteristics of Female Outpatients with uUTI Failing or not Failing Empirically Prescribed Oral Antibiotics

Characteristics	Patients with TF (*n* = 62,873 cases)	Patients without TF (*n* = 313,131 cases)	Std diff (%)*	Incidence of TF (%)
Empiric oral antibiotic prescribed on the index date, *n* (%)				
NTF	21,478 (34.2)	119,008 (38.0)	8.0	15.3
SXT	16,378 (26.0)	79,547 (25.4)	1.5	17.1
β-lactams	16,187 (25.7)	68,017 (21.7)	9.5	19.2
Fluoroquinolones	8710 (13.9)	46,280 (14.8)	2.6	15.8
Fosfomycin	120 (< 5)	279 (< 5)	2.7	30.1
Antibiotics received up to 6 days prior to the index uUTI diagnosis, *n* (%)				
NTF	7148 (11.4)	27,803 (8.9)	8.3	20.5
SXT	6515 (10.4)	23,569 (7.5)	9.9	21.7
β-lactams	17,918 (28.5)	68,731 (21.9)	15.1*	20.7
Fluoroquinolones	6378 (10.1)	22,141 (7.1)	11.0*	22.4
Fosfomycin	84 (< 5)	200 (< 5)	2.2	29.6
Prior non-susceptible pathogen ^‡^, *n* (%)				
Any prior antibiotic non-susceptibility	2803 (4.5)	9886 (3.2)	6.8	22.0
Test for non-susceptibility to NTF	4105 (6.5)	16,157 (5.2)	5.8	20.3
Non-susceptible	675 (16.4)	2074 (12.8)	10.2*	24.6
Susceptible	3308 (80.6)	13,793 (85.4)	12.8*	19.3
Test for non-susceptibility to SXT	4311 (6.9)	16,692 (5.3)	6.4	20.5
Non-susceptible	851 (19.7)	2784 (16.7)	7.9	23.4
Susceptible	3299 (76.5)	13,512 (80.9)	10.8*	19.6
Test for non-susceptibility to β-lactams	4519 (7.2)	17,621 (5.6)	6.4	20.4
Non-susceptible	2311 (51.1)	7984 (45.3)	11.7*	22.4
Susceptible	2140 (47.4)	9393 (53.3)	11.9*	18.6
Test for non-susceptibility to fluoroquinolones	4183 (6.7)	16,478 (5.3)	5.9	20.3
Non-susceptible	632 (15.1)	1770 (10.7)	13.0*	26.3
Susceptible	3463 (82.8)	14,455 (87.7)	14.0*	19.3
Tested for non-susceptibility to fosfomycin	89 (< 5)	345 (< 5)	0.9	20.5
Non-susceptible	< 5 (11)	< 5 (< 5)	0.3	20.0
Susceptible	83 (93.3)	330 (95.7)	10.5*	20.1
Prescribing provider specialty, *n* (%)				
Primary care^§^	44,572 (70.9)	242,303 (77.4)	14.9*	15.5
Emergency medicine/urgent care centers	7079 (11.3)	29,103 (9.3)	6.5	19.6
Obstetrics and gynecology	1953 (< 5)	10,674 (< 5)	1.7	15.5
Urology	1121 (< 5)	6228 (< 5)	1.5	15.3
Infectious disease	585 (< 5)	3536 (< 5)	2.0	14.2
Other^¶^	303 (< 5)	1491 (< 5)	0.1	16.9
Unknown	7787 (12.4)	23,208 (7.4)	16.7*	25.1
Visit type of index uUTI diagnosis, *n* (%)				
Office/clinic patient	27,439 (43.6)	146,238 (46.7)	6.2	15.8
Emergency patient	14,988 (23.8)	54,008 (17.2)	16.4*	21.7
Ambulatory patient services	13,269 (21.1)	75,788 (24.2)	7.4	14.9
Telephone/online	4437 (7.1)	18,952 (6.1)	4.1	19.0
Urgent care	2740 (< 5)	18,145 (5.8)	6.5	13.1
Recurrent UTI, *n* (%)^**^	14,215 (22.6)	53,190 (17.0)	14.1*	21.1
Elixhauser comorbidity score (per unit increase)^††^, mean ± SD	0.7 ± 4.2	0.5 ± 3.7	6.3	–
Diabetes^††^,* n* (%)				
Diabetes mellitus with no HbA1c test	6095 (9.7)	25,249 (8.1)	5.7	19.5
Controlled diabetes mellitus	1532 (2.4)	6506 (< 5)	2.4	19.1
Prediabetes	3975 (6.3)	17,730 (5.7)	2.8	18.3
Obesity^††^, *n* (%)	11,072 (17.6)	47,298 (15.1)	6.8	19.0
Number of hospitalizations prior to index^††^, mean ± SD	0.1 ± 0.3	0.0 ± 0.2	6.7	–
Number of oral ABX prescriptions prior to index^††^, *n* (%)				
0	30,373 (48.3)	177,690 (56.7)	17.0*	14.6
1	13,171 (20.9)	66,683 (21.3)	0.9	16.5
2	7683 (12.2)	33,408 (10.7)	4.9	18.7
3 +	11,646 (18.5)	35,350 (11.3)	20.4*	24.8
History of antibiotic TF	7629 (12.1)	14,845 (< 5)	26.8*	33.9
Antibiotic allergy^††^, *n* (%)	6091 (9.7)	23,948 (7.6)	7.3	20.3

^*^A std diff > 10% was considered a clinically meaningful imbalance between patients with TF than those without TF

^‡^Percentages calculated among patients with ≥ 1 any prior antibiotic susceptibility test for the respective antibiotic drug/class during the baseline period, not including the index uUTI. Percentages do not add up to 100% as some test results were indeterminant

^§^Included healthcare providers in primary care, family medicine, internal medicine, general practice, general pediatrics or clinics

^¶^Included all specialties not encompassed by primary care, urology, infectious disease, obstetrics and gynecology, or emergency medicine/urgent care centers

^**^Defined as a diagnosis of recurrent UTI, identified in clinician notes, or as two UTIs in the past six months or three UTIs in the past 12 months, including the index uUTI diagnosis

^††^Evaluated during the 12-month baseline period (excluding the index date)

HbA1c, hemoglobin A1c; NTF, nitrofurantoin; Std diff, standardized difference; SXT, trimethoprim-sulfamethoxazole; TF, treatment failure; UTI, urinary tract infection; uUTI, uncomplicated urinary tract infection

### Incidence of TF

**Figure 2:**
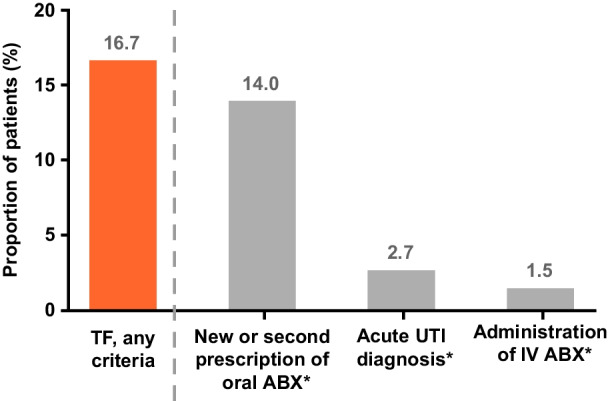
Incidence of TF to Empirically Prescribed Oral Antibiotics Among Female Outpatients with uUTI. *Not mutually exclusive. A patient could have experienced > 1 criterion. ABX, antibiotic; IV, intravenous; TF, treatment failure; UTI, urinary tract infection; uUTI, uncomplicated urinary tract infection

Incidence of TF was 16.7% (*N* = 62,873), primarily driven by the need for a new/repeat prescription of an oral antibiotic (Fig. 2). Mean (SD) time to TF was 7.5 (7.3) days (median: 4.0 days). Results from the sensitivity analysis, when applying a conservative definition of TF, were consistent with the primary analysis (Supplementary Table S1).

Incidence of TF by select baseline characteristics is presented in Tables 1 and 2. Incidence of TF was highest in patients with a history of antibiotic TF (33.9%) or a prescription of fosfomycin on the index date (30.1%) or in the baseline period (29.6%). In patients with any prior antibiotic non-susceptibility, the incidence of TF was 22.1%, and the incidence of TF increased with patient age (≥ 75 years: 20.9%), recurrent UTI (21.1%), and prior oral antibiotic prescriptions (≥ 3 prescriptions: 24.8%).

### Risk Factors for TF

**Figure 3:**
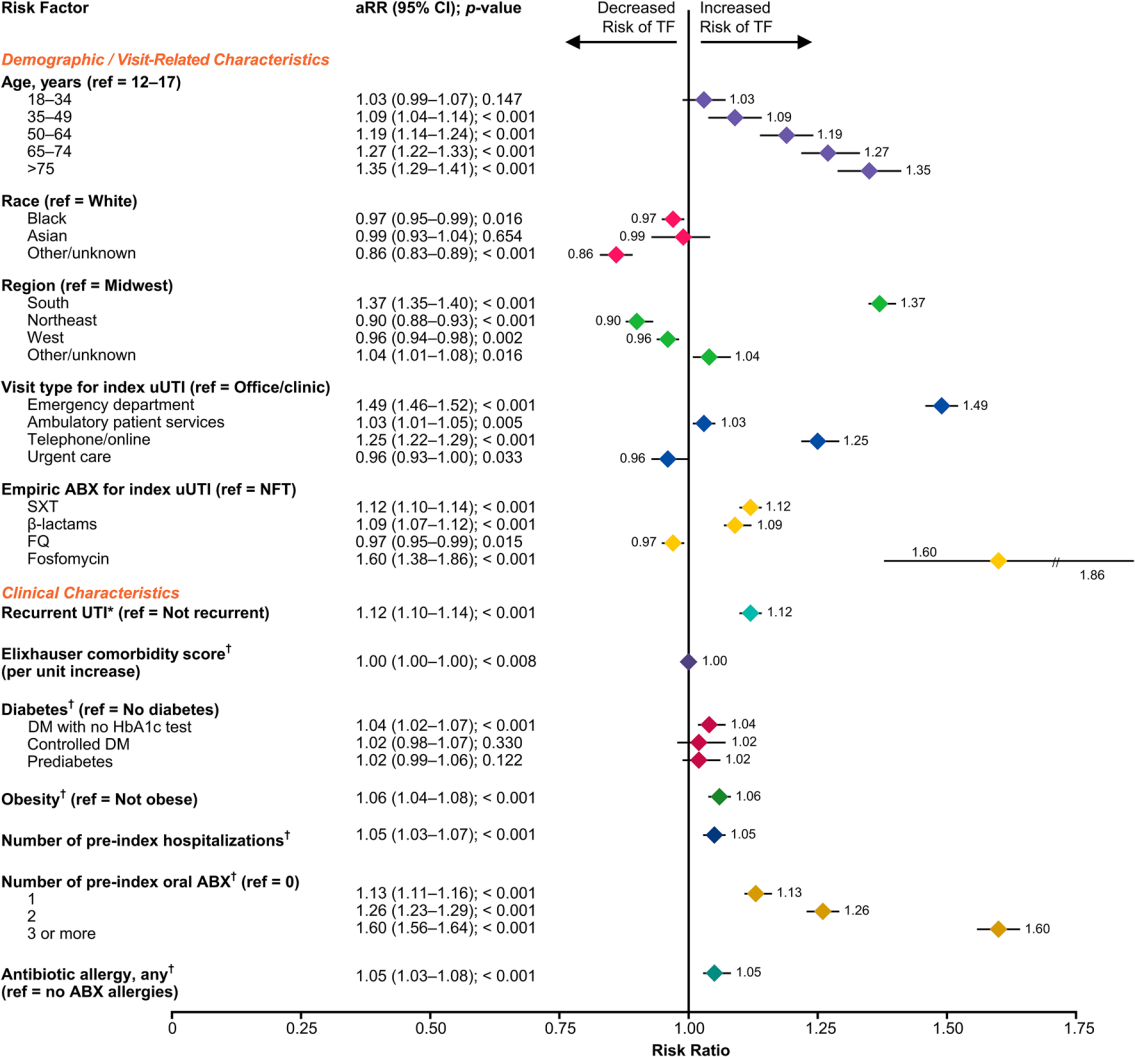
Risk Factors Associated with TF to Empirically Prescribed Oral Antibiotics in uUTI. Risk factors were assessed on the index date unless otherwise specified. Five candidate risk factors (ethnicity, prior fever, prior chills, number of pre-index emergency department visits and any prior ABX non-susceptibility) were not selected by the LASSO model as risk factors for TF. *Defined as a diagnosis of recurrent UTI, identified in clinician notes, or as two UTIs in the past six months or three UTIs in the past 12 months, including the index uUTI diagnosis. ^†^During the 12-month baseline period, not including the index date. ABX, antibiotic; BL, β-lactams; CI, confidence interval; DM, diabetes mellitus; FQ, fluoroquinolones; HbA1c, hemoglobin A1c; LASSO, least absolute shrinkage and selection operator; NTF, nitrofurantoin; ref, reference; RR, risk ratio; SXT, trimethoprim-sulfamethoxazole; TF, treatment failure; UTI, urinary tract infection; uUTI, uncomplicated urinary tract infection.

Seventeen candidate risk factors were included in the LASSO-penalized regression model, of which 12 were selected as risk factors for TF (Fig. 3). Patients who were previously prescribed ≥ 3 oral antibiotics had a 60% higher risk of TF than patients with no prior oral antibiotic prescriptions (aRR 1.60, 95% CI 1.56–1.64); patients previously prescribed one and two oral antibiotics had a 13% (aRR 1.13, 95% CI 1.11–1.16) and 26% (aRR 1.26, 95% CI 1.23–1.29) higher risk of TF, respectively (all *p* < 0.001). Compared to NTF prescription, fosfomycin had the highest risk of TF (aRR 1.60, 95% CI 1.38–1.86),).

Compared to index uUTI diagnosis in the office/clinic, patients diagnosed in the ED had the highest risk of TF (aRR 1.49, 95% CI 1.46–1.52; *p* < 0.001), followed by diagnosis over telephone/online (aRR 1.25, 95% CI 1.22–1.29; *p* < 0.001). Patients residing in the South had higher risk of TF than patients residing in the Midwest (aRR 1.37, 95% CI 1.35–1.40; *p* < 0.001). Older age was consistently associated with a higher risk of TF compared to age 12–17 years, with the highest risk observed in patients aged ≥ 75 years (aRR 1.35, 95% CI 1.29–1.41; *p* < 0.001). Patients with recurrent UTI had an higher risk of TF than those without recurrent UTI (aRR 1.12, 95% CI 1.10–1.14; *p* < 0.001).

Additional clinical characteristics associated with an increased risk of TF included: obesity, increasing number of baseline hospitalizations, antibiotic allergy, and diabetes with no HbA1c test (Fig. 3).

## DISCUSSION

The incidence of TF observed in this study is considerable, with one in every six female outpatients with uUTI experiencing TF to empirically prescribed oral antibiotics. To our knowledge, this study provides the most current and comprehensive estimate of the incidence of TF to empirically prescribed oral antibiotics in female outpatients with uUTI and is the first to shed light on the risk factors of TF in the US. As such, our findings advance the understanding of the risk of TF with current uUTI antibiotic treatment options and highlight the sub-populations at elevated risk for TF, among whom urinalysis may enhance treatment decision-making and mitigate the risk of antibiotic overprescribing in clinical practice.^15^

The incidence of TF observed in this study (16.7%) approaches the upper range of TF rates previously reported in observational studies of female patients with uUTI.^9,23^ Indeed, Franklin et al. (2023)^11^ observed TF in 12.3% of empirically treated female outpatients with uUTI in the US when using the same definition of TF as in this study. The lower incidence of TF reported by Franklin et al. may be driven by the use of administrative claims data, which provide insights into medications dispensed. As the EHR data utilized in this study accounts for antibiotic prescriptions that would not be captured in administrative claims data, it may provide a more complete estimate of the incidence of TF. EHR data provide insights into medications prescribed and therefore, prescriptions of a new or repeat antibiotic treatment are likely indicative of a lack of initial treatment response from the clinician perspective. Moreover, the incidence of TF in this study aligns with other claims-based studies of UTI. For example, Butler et al. (2021) reported a crude TF risk (defined by antibiotic prescription switch/repeat) among premenopausal women with uUTI of 13.6%.^5^ The incidence of TF in this study was 14.0% when defined by a new or repeat oral antibiotic prescription, consistent with Butler et al. despite their inclusion of a younger population (median age: 29–32 years) and use of administrative claims data. Therefore, the incidence of TF in this study is corroborated by prior literature, suggesting that our definition of TF is a reliable approach to estimating this outcome in EHR data, and underscores the limitations of current oral antibiotics in the management of uUTI.

One of the strongest risk factors of TF in this study was prior antibiotic prescriptions. Notably, ≥ 3 prior antibiotic prescriptions within ≤ 12 months of a uUTI imposed a 60% higher risk of empiric TF than no prior antibiotic prescriptions. Similarly, having one prior UTI within six months or two within ≤ 12 months of a uUTI imposed a 12% higher risk of TF than no prior UTIs. These findings highlight the impact of past infections, specifically those that require antibiotic prescriptions or that are UTI-related, on subsequent TF and corroborate guideline recommendations for obtaining a urine culture and susceptibility test in patients with recurrent uUTI.^24,25^ In other studies, prior antibiotic exposure and UTI episodes have been identified as important risk factors for AMR.^26–28^ Although prior antibiotic non-susceptibility was not identified as a risk factor of TF in this study, future research may extend our work by assessing the individual association between non-susceptibility to the empirically prescribed oral antibiotic and subsequent TF in this population. Nevertheless, our findings highlight an implicit association between AMR and TF that provides support for the consideration of local AMR prevalence^8^ to optimally treat uUTI.

Across oral antibiotics prescribed, the incidence of TF was varied and fosfomycin was associated with the highest risk of TF compared to NTF. In a RCT, treatment with fosfomycin was similarly observed to increase the likelihood of clinical TF by 2.35 times.^14^ Fosfomycin is an infrequently used uUTI treatment in the US due to efficacy concerns and high cost.^29^ Accordingly, few patients were prescribed fosfomycin in the present study (< 5%), which may suggest a differential risk profile with respect to TF when other first-line agents are not considered. Prescriptions of SXT and β-lactams were also identified as risk factors of TF relative to NTF. The higher risk of TF associated with β-lactams is not unexpected, given its placement as a second-line agent due to adverse outcome concerns.^8,25^ The higher risk of TF associated with SXT may be driven by increasing rates of non-susceptibility to SXT. Between 2011 and 2019, the prevalence of non-susceptibility to SXT among uropathogenic *Escherichia coli (E. coli)* in the US was consistently ≥ 25%.^30^ SXT is not recommended by IDSA guidelines as first-line therapy when community non-susceptibility rates are ≥ 20%.^8^ Thus, SXT therapy in this study may have been discordant with treatment guidelines.^31^ These findings suggest that the utility of some first-line therapies in the treatment of uUTI may be limited and future research investigating the impact of community non-susceptibility rates on risk of TF are warranted.

Location of care, including healthcare setting and geographic region, was significantly associated with a higher risk of TF in uUTI. An index uUTI diagnosis in the ED increased the risk of TF by nearly 50% versus a diagnosis in the office/clinic. Previous research suggests that inappropriate antibiotic prescribing may contribute to higher risk of TF in the ED than in the office/clinic.^32^ The challenges of managing UTIs in EDs, including limited medical history, limited longitudinal follow-up, and lack of culture and susceptibility results, may further contribute to increased TF.^33^ Residence in the South (versus MidWest) was associated with higher risk of TF, which may be driven by higher rates of antibiotic prescribing and inferior antibiotic stewardship practices in the South than other US regions.^34^ Poor empiric treatment practices are associated with clinical failure, including increased risk of AMR and the need for alternative antibiotics.^35^ A retrospective US study of *E. coli* isolates from female outpatients with uUTI observed that AMR rates are highest in Southern US regions.^30^ The South has also been associated with the highest risk of non-susceptibility to NTF, SXT and fluoroquinolones, when controlling for confounding factors.^28^ Careful consideration of the location of care in empiric treatment decision-making, together with effective antibiotic therapies, may help to reduce inappropriate prescribing in this population.

UTIs are one of the most commonly diagnosed infections in older women, due in part to their compromised immune response, vaginal atrophy, hormonal changes and multiple comorbidities.^36,37^ UTIs are the most common indication for antibiotic prescriptions in this population, with 40–75% of antibiotic use considered inappropriate.^37^ Older age was significantly associated with a higher risk of TF in this study, with a 27% and 35% higher risk in female outpatients aged 65–74 years and ≥ 75 years, respectively, than those aged 12–17 years. In prior literature, the estimated probability of TF in UTI increased by 2% for each decade after 60 years of age.^6,37^ As older patients with uUTI are at higher risk of TF to empirically prescribed oral antibiotics, it is imperative that effective agents are initiated early in the elderly population.

Findings from this study should be interpreted considering some limitations. Neither the study population nor the definition of TF used in this study required a urinalysis- or culture-proven UTI. This approach may have resulted in the inclusion of misdiagnosed patients in the study and overestimation of TF. However, as urine cultures are not systematically collected in real-world clinical practice, nor specifically recommended for the management of non-recurrent uUTI^8^ our findings may be more generalizable to the broader empirically treated uUTI population. A patient may have received an oral/IV antibiotic for a non-uUTI related condition, which may overestimate TF. Nevertheless, findings from the sensitivity analysis suggest that the incidence of TF was consistent when accounting for other infections and possible prophylactic use due to surgeries/procedures. Despite exclusionary efforts, patients with cUTI may be present in the study population due to misclassification. While EHR data provide insights into medications prescribed, they do not indicate if the antibiotic prescription was dispensed and consumed by the patient as prescribed. This limitation may lead to overestimation of TF if patients were noncompliant with their initial empirically prescribed oral antibiotic treatment. Nonetheless, despite this known limitation of EHR data in retrospective, observational studies, the incidence of TF reported in this study largely aligns with published evidence from claims-based studies. Lastly, the requirement of ≥ 12 months of Optum EHR activity post-index may have introduced survival bias. However, given that uUTIs are not associated with high mortality rates,^38^ minimal bias is expected to be introduced by this criterion.

Despite these limitations, key strengths of this study include its reporting of the most current estimate of TF to empirically prescribed oral antibiotics in a large patient population. Moreover, this study is the first to identify risk factors for TF in empirically prescribed female outpatients with uUTI in the US. The dataset utilized was an ideal data source given the large and rich clinical data from which candidate risk factors for TF were derived. The list of candidate risk factors identified in this study were further informed by input from a urology expert.

## CONCLUSION

In this real-world study, we observed an appreciable proportion of female outpatients with uUTI experiencing TF to empirically prescribed, currently available oral antibiotics. Previous antibiotic prescriptions, recurrent UTI, ED setting, and Southern US region were identified as key patient-level risk factors for TF, highlighting the role of prior infections and location of care in subsequent TF. Knowledge of these TF risk factors can be used in clinical practice to inform shared-decision making and supplement existing guidance on appropriate oral antibiotic treatment selection for uUTI in relation to known local antimicrobial susceptibility patterns, urine analysis, and the potential need for urine culture and sensitivity testing. Future studies can provide additional insights by investigating the association between AMR and TF in uUTI, including the impact of community non-susceptibility rates on risk of TF.

## Supplementary Information

Below is the link to the electronic supplementary material.

Supplementary file 1 (DOCX 55.4 KB)

## Data Availability

The datasets generated and analyzed during the current study are not publicly available because they were used pursuant to a data use agreement, but are available from the corresponding author upon reasonable request and permission from Optum.
