# Supplementary material for: Risk Factors for Empiric Treatment Failure in US Female Outpatients with Uncomplicated Urinary Tract Infection: an Observational Study
Source: J Gen Intern Med. 2024 Oct 2;40(4):862–70. doi: 10.1007/s11606-024-09029-6 (PMC11914431; doi:10.1007/s11606-024-09029-6)
Supplement: Supplementary file 1 — (DOCX 55.4 KB) [file 11606_2024_9029_MOESM1_ESM.docx]

**SUPPLEMENTARY MATERIALS**

**Figure S1 Study Design.**


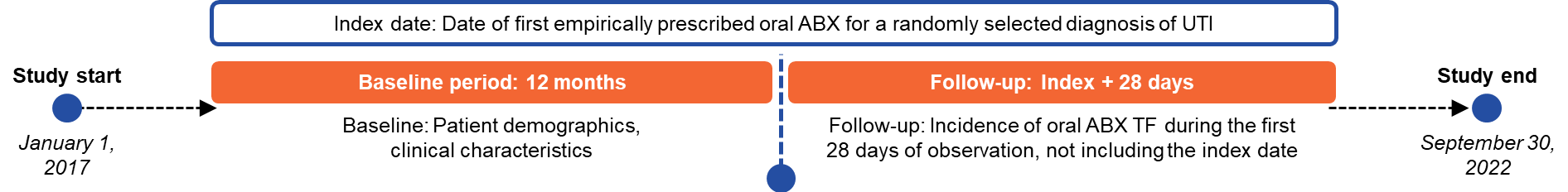


ABX, antibiotics; TF, treatment failure; UTI, urinary tract infection; uUTI, uncomplicated urinary tract infection.

**Study Population**

The study population consisted of female outpatients with uUTI who meet the inclusion and exclusion criteria described below.

**Inclusion Criteria**

The inclusion criteria included:

- Female
- Documented diagnosis code for UTI (International Classification of Diseases, Tenth Edition, Clinical Modification [ICD-10-CM]: N30.00, N30.01, N30.90, N30.91 or N39.0) in an outpatient ambulatory or emergency department (ED) setting between January 1, 2018 and September 30, 2022
  - A randomly selected diagnosis of UTI was defined as the index uUTI diagnosis date
- Prescription for ≥ 1 oral antibiotic agent within ±5 days of the index uUTI diagnosis date
  - Oral antibiotic treatments included nitrofurantoin (NTF), trimethoprim (TMP)-sulfamethoxazole (SXT; separately or in combination), fluoroquinolones (FQ), fosfomycin, and β-lactams
    - Oral fluoroquinolone agents included: levofloxacin, ciprofloxacin and ofloxacin.
    - Oral beta-lactam agents included: amoxicillin, amoxicillin/potassium-clavulanate, ampicillin, cefaclor, cefadroxil, cefdinir, cefditoren, cefditoren-pivoxil, cefixime, cefpodoxime-proxetil, cefprozil, ceftibuten, cefuroxime-axetil, cephalexin, cephradine and loracarbef
  - The date of the initial oral antibiotic treatment for the index uUTI diagnosis was defined as the index date
- ≥ 12 years of age on the index date
- ≥ 12 months of Optum EHR activity prior to the index date
  - The 12 months prior to the index date was defined as the baseline period
- ≥ 12 months of Optum EHR activity after the index date

**Exclusion Criteria**

The exclusion criteria included:

- Antibiotic susceptibility test results of the index uUTI isolate within 14 days prior to or on the index date
- Evidence of a complicated UTI (Sabih A, Leslie SW. Complicated Urinary Tract Infections. StatPearls. StatPearls Publishing Copyright © 2022, StatPearls Publishing LLC.; 2022) during the baseline period or on the index date, unless otherwise specified
  - Pregnancy
    - Pregnancy was identified using 1) ICD-10-CM diagnosis codes, 2) ICD-10 Procedure Coding System (ICD-10-PCS) codes, 3) Current Procedural Terminology (CPT) codes, and 4) “pregnancy” and related terms in clinician notes
  - Diagnosed with urological abnormalities
  - Diagnosed with uncontrolled or complicated diabetes
    - Uncontrolled and complicated diabetes was identified using 1) ICD-10-CM diagnosis codes and 2) hemoglobin A1c (HbA1c) ≥ 8%
  - Diagnosed with severe renal dysfunction
  - Immunosuppressed or treated with immuno-suppressive therapy
  - Urological or nephrological procedures (i.e., catheter, surgery) within 28 days prior to or on the index date and ureteral stent procedure during the baseline period
  - IV antibiotics within 28 days prior to or on the index date
- Hospitalization within 28 days prior to or on the index date
- Resident of a nursing home or long-term care facility during the baseline period or on the index date

**Baseline Characteristics**

The following demographics were assessed on the index date: Age in years, overall and by age groups (12–17, 18–34, 35–49, 50–64, 65–74, ≥ 75 years); geographic region of residence; race; ethnicity; insurance type; provider specialty (primary care, urology, infectious diseases, obstetrics and gynecology, emergence medicine/urgent care centers, other, unknown).

The following microbiology-related characteristics were assessed on the index uUTI diagnosis date (±7 days): Any prior pathogen non-susceptibility; any prior testing for pathogen susceptibility to each of NTF, SXT, FQ, fosfomycin, and β-lactams; the testing results (non-susceptible or susceptible) for those patients tested.

The following clinical characteristics were assessed during the 12-month baseline period, not including the index date (unless otherwise specified): Visit type (urgent care, ambulatory patient services [e.g., hospital outpatient], physician office or clinic, ED, telephone/online), which could be on the index date; empiric antibiotic treatment received on the index date (NTF, SXT, FQ, fosfomycin, and β-lactams); Elixhauser comorbidity score; number of prescriptions of oral antibiotic treatments received (0, 1, 2, 3+); history of oral antibiotic treatment failure (TF) (≥ 1 of the following outcomes ≤ 28 days after the index date: prescription of a new oral antibiotic or a repeat prescription; administration of an IV antibiotic; or primary diagnosis of uncomplicated or complicated UTI in an ED or inpatient setting [excluding the index uUTI diagnosis]); antibiotic resistance of prior infections that are non-UTI specific (NTF, SXT, FQ, fosfomycin, β-lactams, other antibiotics); number of hospitalizations; comorbidities (diabetes and obesity); recurrent UTI (defined as ≥ 3 episodes of a UTI in the past 12 months or ≥ 2 episodes in the past 6 months, including the index uUTI diagnosis); history of antibiotic treatments received in the past 12 months and ≥ 6 days prior to the index uUTI diagnosis date (NTF, SXT, FQ, fosfomycin, β-lactams, other antibiotics); antibiotic allergy.

**Sensitivity Analysis**

A sensitivity analysis was also conducted to evaluate the incidence of TF using a conservative definition of TF. Specifically, the following two alternative definitions of TF were assessed for the oral and IV components of the composite TF definition: (1) prescription of a new or repeat oral antibiotic treatment for uUTI ≤ 28 days following the index date, excluding new or repeat oral antibiotic prescriptions for which a diagnosis of an acute or semi-acute infection (other than a UTI) was documented in the primary position ±5 days of the prescription; (2) administration of an IV antibiotic treatment ≤ 28 days following the index date, excluding IV antibiotic treatments for which a surgical procedure visit was recorded on the same day as the administration. The incidence of TF was evaluated using each alternative definition separately and in combination.

**Table S1 Incidence of Treatment Failure to Initial, Empirically Prescribed Oral Antibiotic Treatment among Female Outpatients with uUTI* – Sensitivity Analysis.**

| **Incidence** | **Outpatient uUTI cohort**  ***N* = 376,004** |
| --- | --- |
| ***Original analysis*** | |
| **TF^†^, *n* (%) [95% CI]^§^** | 62,873 (16.7) [16.6, 16.8] |
| [A] New or repeat prescription of oral antibiotic treatment^‖^ | 52,522 (14.0) [13.9, 14.1] |
| [B] Acute UTI diagnosis^¶^ | 10,244 (2.7) [2.7, 2.8] |
| [C] Administration of IV antibiotic treatment^#^ | 5528 (<5) [1.4, 1.5] |
| ***Alternative definitions of [A]** and [C]^††^*** | |
| **TF^†^, *n* (%) [95% CI]^§^** | 58,800 (15.6) [15.5, 15.8] |
| New or repeat prescription of oral antibiotic treatment^‖,^** | 49,179 (13.1) [13.0, 13.2] |
| Acute UTI diagnosis^¶^ | 10,244 (<5) [2.7, 2.8] |
| Administration of IV antibiotic treatment^#,††^ | 3808 (<5) [1.0, 1.0] |
| ***Alternative definitions of [A]*** ***^†^ only*** | |
| **TF^†^, *n* (%) [95% CI]^§^** | 59,973 (16.0) [15.8, 16.1] |
| New or repeat prescription of oral antibiotic treatment^‖,^** | 49,179 (13.1) [13.0, 13.2] |
| Acute UTI diagnosis^¶^ | 10,244 (<5) [2.7, 2.8] |
| Administration of IV antibiotic treatment^#,††^ | 5528 (<5) [1.4, 1.5] |
| ***Alternative definitions of [C]^††^ only*** | |
| **TF^†^, *n* (%) [95% CI]^§^** | 61,747 (16.4) [16.3, 16.5] |
| New or repeat prescription of oral antibiotic treatment^‖,^** | 52,522 (14.0) [13.9, 14.1] |
| Acute UTI diagnosis^¶^ | 10,244 (<5) [2.7, 2.8] |
| Administration of IV antibiotic treatment^#,††^ | 3808 (<5) [1.0, 1.0] |

*Incidence of TF was evaluated over the first 28 days of the observation period following the index date (i.e., date of first empirically prescribed oral antibiotic treatment for one randomly selected index uUTI diagnosis). ^†^Patients may have met multiple definitions of TF. Therefore, categories of TF are not mutually exclusive and do not sum to the total number of patients with TF. ^§^The Clopper-Pearson Exact method was used to calculate 95% confidence intervals for a binomial proportion. ^‖^Oral antibiotic treatments included NTF, SXT, fluoroquinolones, fosfomycin and β-lactams. ^¶^Acute UTI was defined as having a primary diagnosis of uncomplicated or complicated UTI in an acute care setting (i.e., inpatient or emergency department). ^#^IV antibiotics were identified using HCPCS codes. **The alternative definition of [A] excluded new or repeat oral antibiotic prescriptions for which a diagnosis of an acute or semi-acute infection (other than a UTI) was documented in the primary position ±5 days of the prescription from the definition of TF. ^††^The alternative definition of [C] excluded IV antibiotic treatments for which a surgical procedure visit was recorded on the same day as the administration of the IV antibiotic from the definition of TF.

HCPCS, Healthcare Common Procedure Coding System; IV, intravenous; Q1, first quartile; Q3, third quartile; SXT, trimethoprim-sulfamethoxazole; TF, treatment failure; UTI, urinary tract infection; uUTI, uncomplicated urinary tract infection.
